# Ink for the Million

**Published:** 1852-11

**Authors:** W. H. Pile

**Affiliations:** Philadelphia


*Ink for the Million.*

To the Editor of the American Journal of Pharmacy: The following formula for making a very *superior ink* is not generally known. The facility of its preparation, and its almost incredible cheapness, (about two cents a gallon,) render it worthy a place in your Journal.

℞ 12 oz. avoird. Ext. Logwood,
½ oz. " Bichromate Potash,
5 gallons water;

Dissolve the ingredients separately in water and mix them together, and in a short time the ink will be fit for use.

An analysis of the above would be very desirable.

As an instance of the very great coloring property of hæmatoxylon, I have found that 1-100th of a grain dissolved in 4,000-000 times that quantity of water, will be tinged a fine pink color by the addition of a little aqua ammonia. Yours truly,

W. H. PILE.

*Philadelphia, Sept.* 13, 1852.

